# Supplementary figures and images for: bayroot: Bayesian sampling of HIV-1 integration dates by root-to-tip regression
Source: Virus Evol. 2022 Dec 21;9(1):veac120. doi: 10.1093/ve/veac120 (PMC9825830; doi:10.1093/ve/veac120)

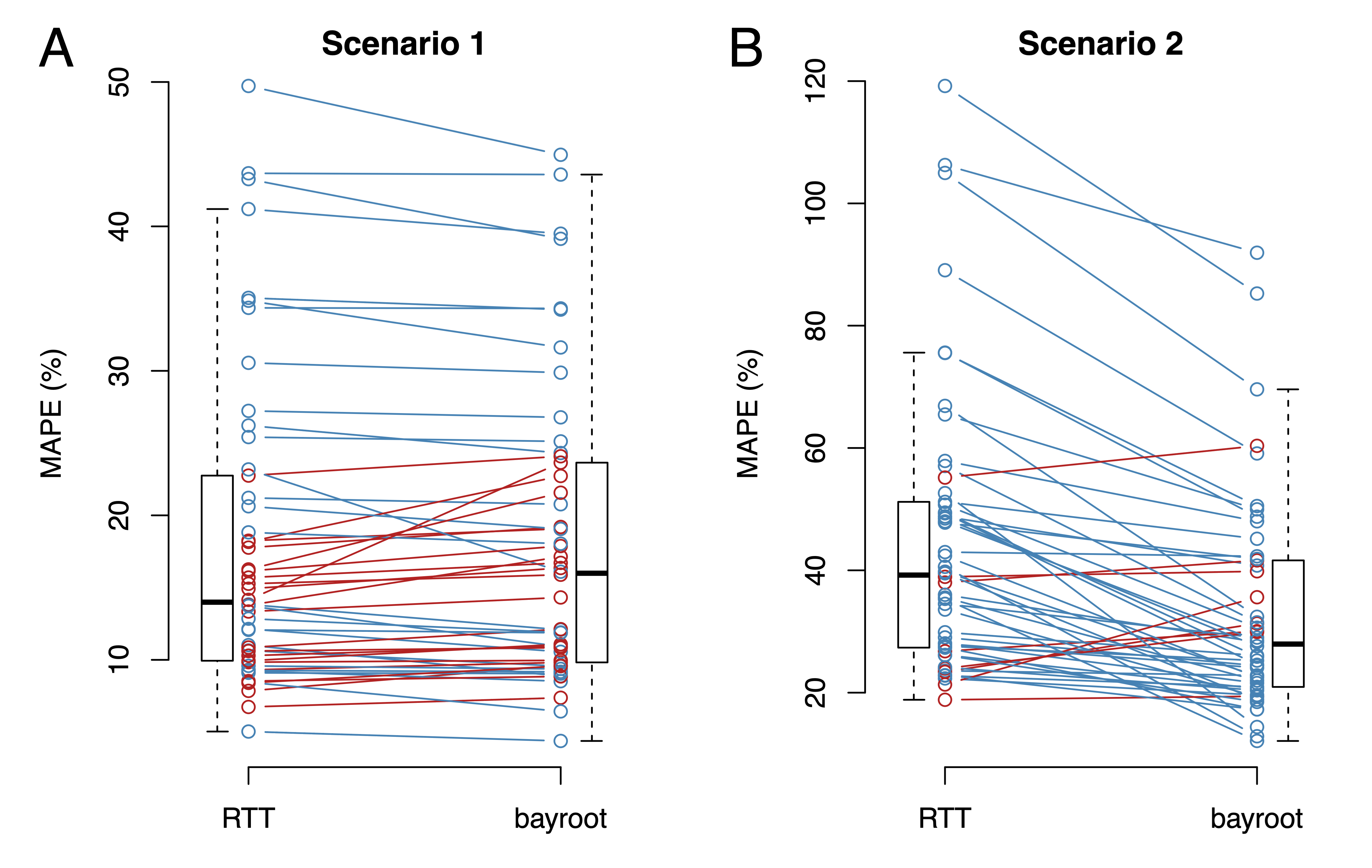

Supplement: veac120_Supp [file veac120_supp.zip › FigureS1.tiff]

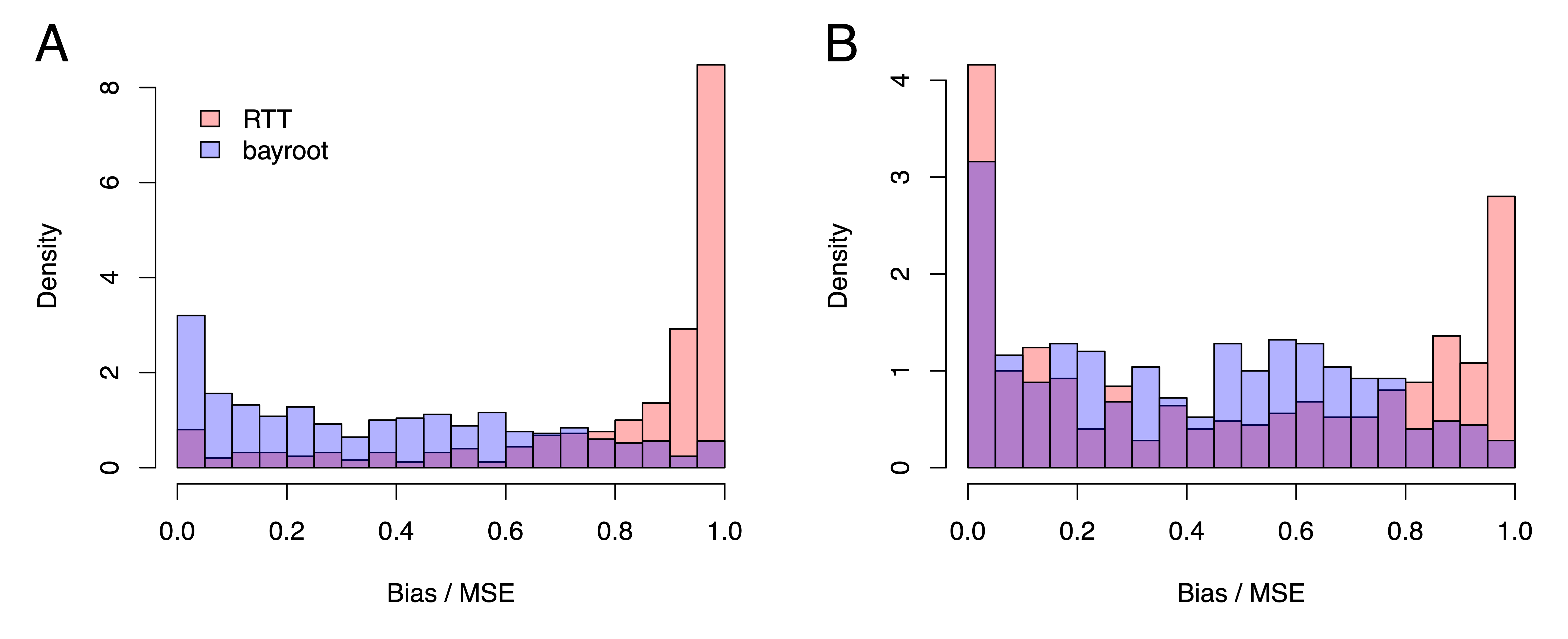

Supplement: veac120_Supp [file veac120_supp.zip › FigureS2.tiff]
